# Impact of Imaging and Pharmacological Treatment Strategies in Refractory Ventricular Tachycardia in Critically Ill Patients: A Systematic Review

**DOI:** 10.7759/cureus.76641

**Published:** 2024-12-30

**Authors:** Paulina Elizabeth Cisneros Clavijo, Alexis Agustin Dunay Silva, John Manuel Dorado Ramírez, Juan Felipe Perez Correa, Yesith Mauricio Montenegro Cadena, Luis Alberto Martínez Arelio, Adriana Viviana Viñan Andino, Daniel Ricardo Cortes Sanchez, Edgard Andres Ramirez Castaño

**Affiliations:** 1 Endovascular Surgery, Enrique Garcés Hospital, Quito, ECU; 2 Hemodynamics, General and Interventional Cardioangiology, Pontificia Universidad Católica del Ecuador, Quito, ECU; 3 Emergency Medicine, Hospital Barros Luco Trudeau, San Miguel, CHL; 4 Internal Medicine, Instituto Mexicano del Seguro Social, San Luis Potosí, MEX; 5 Medicine, Universidad del Rosario, Bogotá, COL; 6 Medicine, Universidad Cooperativa de Colombia, Pasto, COL; 7 Medicine, Centro de Integración para Adictos y Familiares, Monte Fénix, Mexico City, MEX; 8 Medicine, Ministry of Public Health, Ecuador, Quito, ECU; 9 Medicine, Universidad Surcolombiana, Neiva, COL; 10 Medicine, Hospital Universitario Hernando Moncaleano Perdomo, Neiva, COL; 11 Medicine, Universidad Santiago de Cali, Cali, COL

**Keywords:** antiarrhythmic drugs, critical illness, icd interventions, imaging techniques, pharmacological treatments, refractory ventricular tachycardia

## Abstract

Ventricular tachycardia (VT) is a life-threatening arrhythmia often leading to sudden cardiac death, particularly in critically ill patients. Refractory VT, characterized by recurrent episodes requiring intervention, poses unique challenges for management, necessitating advanced diagnostic and therapeutic strategies. This systematic review evaluates the impact of imaging and pharmacological treatments in managing refractory VT in critically ill patients. A systematic literature search was conducted using keywords such as "refractory ventricular tachycardia", "critical illness", "imaging techniques", "pharmacological treatments", "antiarrhythmic drugs", "ICD interventions", and "non-invasive therapy". Databases searched included PubMed, Google Scholar, and Cochrane Library, identifying 1590 publications. After screening, 11 studies meeting the inclusion criteria were included in this review. Oral procainamide significantly reduced VT episodes but caused severe side effects in certain patients. Noninvasive interventions such as transcutaneous magnetic stimulation (TcMS) and noninvasive electrophysiology-guided radioablation reduced VT burden and antiarrhythmic drug (AAD) use, with TcMS decreasing VT episodes in the sham group (P < 0.001). Stereotactic body radiation therapy (SBRT) and stereotactic arrhythmia radiotherapy (STAR) reduced VT episodes. Ultrasound-guided stellate ganglion blockade decreased VT episodes (P < 0.001) within 24 hours. Catheter ablation improved composite outcomes, including ICD shocks and heart failure hospitalizations, compared to AAD therapy. Quality of life significantly improved with noninvasive therapies, though SBRT presented rare complications like pneumonitis. Imaging and pharmacological interventions effectively reduce VT burden and ICD interventions while showing varying safety profiles. However, the limited sample sizes, short follow-up durations, and heterogeneity across studies highlight the need for further high-quality research to establish long-term efficacy and safety.

## Introduction and background

Ventricular tachycardia (VT) is a wide complex tachycardia, defined as three or more consecutive beats at a rate of more than 100 per minute, arising from the ventricle [1,2]. VT is a severe form of arrhythmia, and this rhythm is the cause of most out-of-hospital cardiac arrests in the United States [3]. VT is responsible for 8% of wide-complex tachycardia episodes [1]. The incidence of VTs has rapidly declined since the broad adoption of acute revascularization in patients with myocardial infarction and occurred in 2.2% of all ICU patients in a multicentre cohort study published in 2008 [4]. Importantly, it has been shown that critically ill patients with both supraventricular and ventricular arrhythmias have an increased mortality risk [5].

Refractory VT is a type of arrhythmia that is resistant to pharmacologic therapy and is potentially fatal, causing sudden cardiac death. It does not respond to normal therapy interventions in critically ill patients and is particularly challenging in that the common etiology of this phenomenon includes a reversal of electrolyte imbalance, ischemia, or toxicity to a particular drug [6]. Furthermore, these patients are usually presented with multiple co-morbid conditions that increase diagnostic and therapeutic challenges as well as individual medical management plans. Refractory VT can only be managed using a combination of diagnostic tools and therapeutic techniques, making it important that they be used in combination [7]. 

Antiarrhythmic medications like amiodarone and lidocaine are central to management; amiodarone is administered as a 300 mg IV bolus during cardiac arrest, followed by 150 mg, or as 150 mg in 100 mL D5W (dextrose 5% in water) over 10 minutes for stable VT, with subsequent infusion at 1 mg/minute for six hours, while lidocaine is given as an initial bolus of 1-1.5 mg/kg intravenous/intraosseous (IV/IO), repeatable at 0.5-0.75 mg/kg every 5-10 minutes, with a maintenance infusion of 1-4 mg/minute, not exceeding 3 mg/kg in an hour [8]. Beta-blockers like esmolol, administered as a continuous infusion at 50-300 mcg/kg/minute, reduce sympathetic tone, increase the fibrillation threshold, and improve outcomes in recurrent VT. Imaging-based treatments offer innovative options for managing refractory VT by enhancing precision and enabling noninvasive or minimally invasive interventions [9-11]. Techniques like transcutaneous magnetic stimulation (TcMS), computational ECG mapping with respiratory gating, and cardiac CT (CCT) integrated with electroanatomical mapping (EAM) improve the localization of arrhythmogenic foci. Advanced modalities such as stereotactic arrhythmia radiotherapy (STAR), stereotactic body radiation therapy (SBRT), and noninvasive electrophysiology-guided cardiac radioablation deliver targeted therapy to suppress arrhythmias in resistant cases, providing promising alternatives to traditional treatments [10,11].

Kovacs et al. conducted a systematic review evaluating the safety and efficacy of STAR for treating therapy-refractory VT or ventricular fibrillation (VF) in patients with structural heart disease [12]. Their findings demonstrated that STAR significantly reduces the burden of sustained VT/VF in the short term, though recurrences are common, likely due to the low radiation dose used in the studies reviewed. Despite its potential, there was a high rate of adverse effects (81%), though no treatment-related deaths were reported [12]. Importantly, this review highlights STAR as a promising option but does not address broader strategies for managing refractory VT in critically ill patients. No systematic review has comprehensively examined the combined impact of imaging and pharmacological treatment strategies in this context. To fill this critical literature gap, we conducted a systematic review exploring these integrative approaches in the management of refractory VT. This study seeks to comprehensively assess imaging and pharmacological interventions for refractory VT. By analyzing their combined efficacy and safety, this review aims to identify evidence-based management strategies that improve patient outcomes while addressing existing gaps in the literature.

## Review

Methodology

Study Design

This systematic review was conducted in accordance with the Preferred Reporting Items for Systematic Reviews and Meta-Analyses (PRISMA) guidelines.

Sample Selection

A comprehensive search strategy was employed to identify relevant studies published in English. Databases such as PubMed, Google Scholar, and Cochrane Library were searched. The database search employed specific Boolean operator combinations, such as ("ventricular tachycardia" [MeSH Terms] OR "ventricular tachycardia" OR "refractory ventricular tachycardia" OR "intractable ventricular tachycardia") AND ("critically ill" [MeSH Terms] OR "intensive care" OR "critical illness" OR "ICU") AND (("cardiac imaging techniques" [MeSH Terms] OR "cardiac MRI" OR "cardiac CT" OR "echocardiography" OR "PET") OR ("anti-arrhythmia agents" [MeSH Terms] OR "antiarrhythmic drugs" OR "amiodarone" OR "lidocaine" OR "mexiletine" OR "beta-blockers" OR "propranolol" OR "esmolol" OR "metoprolol" OR "sympatholytic agents" OR "sedation" OR "anesthetic drugs" OR "vasopressors")) AND ("mortality" OR "survival rate" OR "ventricular arrhythmia recurrence" OR "treatment outcome" OR "adverse effects") AND ("clinical trial"[Publication Type] OR "randomized controlled trial" OR "cohort studies" OR "retrospective studies"), to ensure comprehensive coverage of relevant literature. In PubMed, Medical Subject Headings (MeSH) terms were utilized to enhance the precision of the search, including terms such as “Ventricular Tachycardia", “Cardiac Imaging Techniques", and “Antiarrhythmic Agents".

Inclusion Criteria

Eligible studies were required to meet several criteria, including randomized controlled trials (RCTs), cohort studies, and clinical trials, that focused on imaging and/or pharmacological treatments for refractory VT or VT storm in human subjects. Only studies published in English were considered. The timeframe for inclusion was restricted to publications from the last 10 years, chosen to capture recent advancements while balancing the relevance of older, seminal studies on the long-term management of VT. Studies had to report on key outcomes such as VT recurrence, implantable cardioverter-defibrillator (ICD) interventions, antiarrhythmic drug use, and treatment-related complications (e.g., device malfunction, drug toxicity). Only articles published in peer-reviewed journals with clear methodologies and data relevant to the study objectives were included.

Exclusion Criteria

The exclusion criteria for this review encompassed publications not written in English, as well as articles categorized as review articles, editorials, or conference abstracts. Research that did not specifically address refractory VT or VT storm was excluded. Additionally, studies that did not evaluate imaging or pharmacological treatments were deemed ineligible. Non-human studies were also excluded. Furthermore, studies that combined imaging or pharmacological interventions with non-traditional therapies, such as gene therapy or advanced cardiac devices, were omitted to maintain a focus on conventional treatment approaches for refractory VT.

Study Selection Process

The studies identified relevant articles based on the titles and abstracts of the articles obtained from the searches. Abstracts and indexes were initially scanned to ensure they satisfied the inclusion and exclusion criteria for the studies. The screening was conducted using the Rayyan tool (Rayyan Systems, Inc., Cambridge, Massachusetts, United States) and EndNote™ (Clarivate Plc, Philadelphia, United States). Inter-rater agreement was quantified using Cohen’s kappa to assess the consistency between reviewers.

Data Extraction

Data from the included studies were extracted using a pre-designed data extraction form developed at the beginning of the review process. Key data points included study features (authors, year of publication, study type), patient characteristics (age, sex, comorbidities), and details of imaging or pharmacological interventions (type of treatment, VT burden, ICD shocks, anti-arrhythmic drug use, adverse effects, mortality). In cases of missing or incomplete data, attempts were made to contact study authors for clarification. Additionally, the data extraction form was pre-tested on a subset of articles to ensure consistency and accuracy.

Quality Assessment

The methodological quality of the included studies was assessed using the Newcastle-Ottawa Scale (NOS) for observational studies and the Cochrane Collaboration tools for randomized controlled trials.

Data Analysis

Results were synthesized qualitatively due to the heterogeneity of interventions and outcomes. Where applicable, findings were summarized narratively under key outcome categories such as reduction in VT episodes, ICD interventions, antiarrhythmic drug (AAD) use, safety, composite outcomes, and quality of life. Meta-analysis was not conducted due to the variation in study designs and outcomes. However, statistical significance reported in individual studies (e.g., P-values) was highlighted to provide insight into the effectiveness of the interventions.

Results

**Figure 1:**
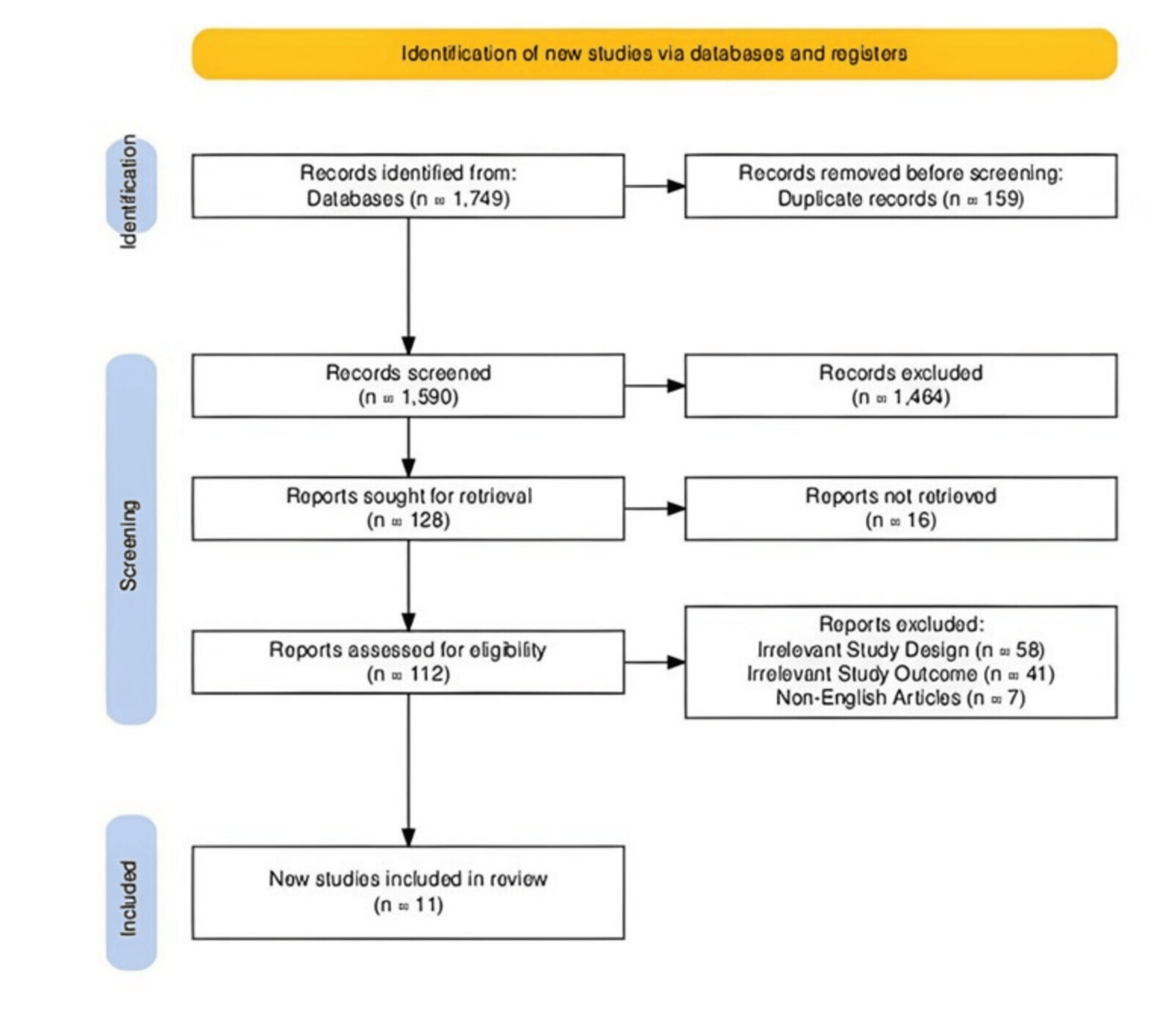
PRISMA Flow Diagram PRISMA: Preferred Reporting Items for Systematic Reviews and Meta-Analyses

The initial literature search conducted using Google Scholar, Cochrane Library, and Pubmed identified 1749 publications. After a meticulous review of titles and abstracts, 128 articles were shortlisted for further evaluation. Full-text versions of these articles were obtained and screened. Studies that did not meet the inclusion criteria, such as those not explicitly addressing refractory VT or not focusing on critically ill patient populations, were excluded. Following the screening process, 11 studies were ultimately included in this systematic review (Figure 1).

Study Characteristics

Six cohort studies (three prospective, one retrospective, one prospective pre-post intervention, and one long-term follow-up study), four RCTs, and one clinical trial comprised the 11 papers that made up this systematic review. The number of participants in the cohort studies varied from six to 34, and the patient groups included a variety of conditions, such as treatment-refractory ventricular arrhythmias and refractory VT. The studies had participant counts ranging from six to 259 people. 

The research assessed a variety of intervention modalities, such as pharmaceutical therapies (like oral procainamide), sophisticated imaging-based methods (including computational ECG mapping and SBRT), catheter ablation, and stellate ganglion blocking (SGB). These procedures aimed to reduce VT or VF episodes, enhance cardiac function, decrease ICD shocks or anti-tachycardia pacing (ATP), and assess their safety and effectiveness, among other goals.

Stereotactic arrhythmia radiation and cardiac CT combined with electroanatomical mapping are two notable imaging and mapping approaches that significantly improved results and increased accuracy. The RCTs and cohort designs demonstrated the effectiveness of several therapies in lowering the incidence of VA episodes, ICD interventions, and total arrhythmia burden. The majority of the techniques also showed good safety profiles. In the complete care of ventricular arrhythmias, this review emphasizes the increasing significance of both invasive and non-invasive approaches.

Reduction in VT Episodes

Significant decreases in VT episodes were shown by a number of therapies. The median number of VT/VF episodes decreased from 19 to 5.5 (P < 0.05) when oral procainamide was taken [13]. VT episodes were also considerably decreased by transcutaneous magnetic stimulation (TcMS), with a mean of 4.5 episodes in the TcMS group against 10.7 in the sham group (P < 0.001) [14]. In a similar vein, VT episodes decreased by 67% with STAR, with a median reduction of 87% [15]. According to Weight et al, SBRT significantly decreased defibrillator shocks (60%) and ATP (39%) while also reducing VT occurrences by 59% [16]. With 94% of patients demonstrating a decrease in VT or PVC load, noninvasive electrophysiology-guided cardiac radioablation produced a notable reduction in VT occurrences from 119 to 3 (P < 0.001) [17]. Last but not least, VA episodes dramatically decreased from 5.5 to 0 (P < 0.001) after 24 hours with ultrasound-guided bilateral SGB, with 45% of patients reporting total suppression for 48 hours [18].

Reduction in ICD Interventions and Therapy Discontinuation

In the study by Toniolo et al., the use of oral procainamide significantly reduced ICD interventions and ventricular arrhythmias [13]. However, 8.8% of patients experienced severe side effects, including dyspnea or hypotension, which led to therapy discontinuation. These side effects highlight the need for careful monitoring when using pharmacological treatments in critically ill patients.

Defibrillator Shocks

Defibrillator shocks decreased from 23 ± 12 to 0.67 ± 1.0 (P < 0.001) in computational ECG mapping and respiratory gating, demonstrating improvements in radiation delivery accuracy [19]. Likewise, defibrillator shocks and VT episodes decreased as a result of STAR. A 75% reduction in the utilization of defibrillator shocks was achieved by noninvasive electrophysiology-guided cardiac radioablation, highlighting the potential of these therapies to lower the frequency of shocks and enhance patient outcomes [15-17].

AAD Use

According to Markman et al., TcMS significantly decreased the need for AADs; on average, the TcMS group needed 0.9 AADs, whereas the sham group needed 1.8 (P = 0.001) [14]. The usage of dual antiarrhythmic medications was also significantly reduced by noninvasive electrophysiology-guided cardiac radioablation going from 59% to 12% (P = 0.008) [17]. Moreover, SBRT resulted in a decrease in ATP and shocks, indicating a possible reduction in the requirement for AADs as part of an overall treatment approach [16].

Safety and Side Effects

The therapies have different safety profiles. 8.8% of patients who took oral procainamide had significant adverse effects, such as hypotension or dyspnea, which caused them to stop their treatment [13]. According to Markman et al., TcMS was well tolerated and had few side effects [14]. With just one incidence of hoarseness documented, ultrasound-guided bilateral SGB experienced few side effects [18]. However, four patients had pneumonitis after receiving SBRT underscoring the need for more research on the treatment's long-term safety [16]. According to Robinson et al., noninvasive electrophysiology-guided cardiac radioablation was usually safe, with only minor short-term concerns and no serious side effects noted [17].

Composite Outcomes (CV Death, ICD Shock, HF Hospitalization)

The risk of composite adverse outcomes was much lower in the catheter ablation group as compared to the AAD group, according to studies. The ablation group had a lower proportion of unfavorable outcomes (28.2%) than the AAD group (46.6%, P = 0.021) in a study by Arenal et al. [20]. The catheter ablation group also had a lower composite result of heart failure hospitalizations, ICD shocks, and CV mortality (59.1%) than the escalated AAD group (68.5%, P = 0.04), according to Sapp et al. [21].

The characteristics of the included studies are symmarized in Table 1.

**Table 1:** Characteristics and results of the studies reviewed ICD: implantable cardioverter defibrillator, VT: ventricular tachycardia; VF: ventricular fibrillation; TcMS: transcutaneous magnetic stimulation; AAD: antiarrhythmic drug; TMS: transcutaneous magnetic stimulation; STAR: stereotactic arrhythmia radiotherapy; SBRT: stereotactic body radiation therapy; ATP: anti-tachycardia pacing; EAM: electroanatomical mapping; CCT: cardiac CT; SGB: stellate ganglion blockade

N°	Authors	Year	Study Design	Number of Participants	Intervention	Outcomes Assessed	Results	Conclusion
1	Toniolo et al. [13]	2021	Retrospective Cohort Study	34 (32 male, 94.1%)	Pharmacological (Oral Procainamide)	ICD interventions, VT/VF episodes, Therapy discontinuation	Median number of VT/VF episodes decreased significantly from 19 [7.5-30] to 5.5 [0.75-30], P < 0.05. 8.8% of patients (3 patients) experienced severe side effects (dyspnea or hypotension), leading to discontinuation of therapy.	Oral procainamide significantly reduced ICD interventions and ventricular arrhythmias in patients with recurrent VT/VF, demonstrating a tolerable side effect profile for most patients. Further studies on long-term outcomes and side effects are recommended.
2	Markman et al. [14]	2022	Double-blind, Sham-controlled Randomized Clinical Trial	26 (20 male, 77%)	Imaging: Transcutaneous Magnetic Stimulation (TcMS), Sham Stimulation	VT recurrence, VT burden (episodes), AADs, Safety (device effects)	VT recurred in 29% of the TcMS group vs 58% in the sham group (P = 0.20). In the 72 hours after randomization, the TcMS group had 4.5 episodes of VT (mean) vs 10.7 episodes in the sham group (P < 0.001). The TcMS group required fewer AADs after 24 hours (mean 0.9 vs 1.8, P = 0.001).	TMS is safe and may reduce the burden of VT episodes in patients with VT storms, suggesting its potential as a non-invasive treatment for stabilizing patients in VT storms.
3	Ho et al. [19]	2021	Prospective Cohort	6	Imaging: Computational ECG Mapping, Respiratory Gating	VT Exit Sites Mapping, Radiation Delivery Precision, Implantable Cardioverter-Defibrillator Shocks	100% colocalization of computational ECG mapping to prior invasive mapping Significant reduction in planning target volume (71 ± 7 cc vs 153 ± 35 cc, P < .01) Decreased defibrillator shocks from 23 ± 12 to 0.67 ± 1.0 (P < .001)	Computational ECG mapping and respiratory gating improve the precision, safety, and efficacy of stereotactic ablative radiotherapy in refractory VT, leading to reduced defibrillator shocks.
4	Van et al. [15]	2023	Prospective, Pre-Post Intervention Study	6	Imaging: STAR	Reduction in VT episodes, Cardiac function, Pulmonary function	67% (4/6) of patients had ≥50% reduction in VT episodes, a median reduction of 87%. No reduction in LV ejection fraction or pulmonary function.	STAR is effective in reducing VT episodes by ≥50% in patients with therapy-refractory VT, with no significant adverse effects on cardiac or pulmonary function.
5	Wight et al. [16]	2021	Long-term follow-up study	14	Imaging: SBRT	Total duration of VT, Frequency of ATP, Frequency of shocks, Complications (e.g., pneumonitis)	59% reduction in VT, 39% reduction in ATP, 60% reduction in shocks; 4 patients developed pneumonitis	SBRT provides modest improvement in managing refractory VT with significant reductions in VT, ATP, and shock events. However, pneumonitis was a complication in some patients. Further randomized controlled trials are needed to establish its efficacy long-term.
6	Robinson et al., [17]	2019	Phase I/II Trial	19	Imaging: Noninvasive Electrophysiology-Guided Cardiac Radioablation (SBRT)	Reduction in VT episodes Reduction in PVC burden Quality of life (SF-36)	Median VT episodes reduced from 119 to 3 (P<0.001); 94% of patients had reduced VT or PVC burden, 75% reduction in VT or PVC burden for 89% of patients, 89% survival at 6 months, 72% at 12 months; Dual antiarrhythmic use reduced from 59% to 12% (P=0.008), Improvement in 5/9 SF-36 domains	Noninvasive electrophysiology-guided cardiac radio ablation markedly reduced VT burden, with modest short-term risks. It led to a reduction in antiarrhythmic drug use and improved quality of life.
7	Conte et al., [22]	2021	Prospective Cohort	19	Imaging: CCT integrated with EAM	Myocardial fibrosis detection in refractory VT Diagnostic accuracy	94.1% diagnostic accuracy for detecting myocardial fibrosis (CCT vs EAM) on a per-segment basis; Successful live integration of CCT with EAM in all patients	CCT integration with EAM is effective and accurate for identifying myocardial fibrosis in patients with refractory VT and contraindications to MRI, aiding in better treatment strategies for this challenging condition.
8	Fudim et al. [18]	2020	Prospective cohort study	20 (Patients with treatment-refractory ventricular arrhythmias)	Ultrasound-guided bilateral SGB	Reduction in VA episodes and defibrillation events within 48 hours	VA episodes reduced from 5.5 (IQR: 2.0-15.8) to 0 (IQR: 0-3.8) in 24 hours (p < 0.001). Defibrillation events decreased from 2.5 (IQR: 0-10.3) to 0 (IQR: 0-2.5) (p = 0.002). Complete suppression for 48 hours in 45%; no recurrence until discharge in 20%. Minimal complications (1 hoarseness case).	SGB significantly reduces VA burden with high safety, achieving complete suppression in nearly half of patients.
9	Arenal et al. [20]	2022	Prospective, multicenter, randomized trial	144 (ICD patients with ischemic cardiomyopathy and symptomatic VT)	Catheter ablation vs antiarrhythmic drugs (AAD: amiodarone ± beta-blockers)	Composite of CV death, ICD shock, HF hospitalization, or severe complications	Primary outcome: 28.2% in ablation group vs 46.6% in AAD group (HR: 0.52; p = 0.021), driven by fewer severe complications (9.9% vs 28.8%, HR: 0.30; p = 0.006). HF hospitalization rates were slightly lower with ablation; cardiac mortality showed no difference.	Catheter ablation outperformed AAD for reducing severe complications and composite outcomes.
10	Sapp et al. [21]	2016	Multicenter, randomized, controlled trial	259 (ICD patients with ischemic cardiomyopathy and recurrent VT)	Catheter ablation with continued AAD vs escalated AAD therapy	Composite of death, VT storm, or appropriate ICD shock	Primary outcome: 59.1% in ablation group vs 68.5% in AAD group (HR: 0.72; p = 0.04). Mortality rates similar. Ablation group experienced 2 cardiac perforations, 3 bleeding cases; AAD group had 2 pulmonary toxicity deaths, 1 hepatic dysfunction death.	Ablation reduced composite outcomes compared to escalated AAD in recurrent VT patients.
11	Deyell et al. [23]	2022	Post-hoc analysis of randomized trial (VANISH)	259 (ICD patients with VT and prior myocardial infarction)	Catheter ablation vs escalated AAD therapy	Death, VT storm, ICD shock; influence of VT cycle length and electrical storm	Slow VT patients had worse outcomes with AAD therapy (HR: 1.7; p = 0.04), especially with baseline amiodarone (HR: 2.22). Ablation trended toward better outcomes for electrical storm patients on amiodarone. No difference for electrical storm across interventions.	Ablation showed better outcomes for slow VT; electrical storm outcomes were comparable across groups.

Quality of Life

According to Robinson et al., noninvasive electrophysiology-guided cardiac radioablation was linked to notable improvements in quality of life, with gains in five of the nine SF-36 quality-of-life areas [17]. Noninvasive therapies are a viable choice for treating individuals with refractory VT because of these advancements, which imply that they improve general health in addition to reducing clinical symptoms. 

The quality of the included studies was assessed using the NOS, with scores ranging from 7 to 9, indicating moderate to high quality. Studies by Toniolo et al. [13], Ho et al. [19], van der Ree et al. [15], Conte et al. [22], and Fudim et al. [18] achieved the maximum score of 9, reflecting their robust design, including representativeness of cohorts, adequate ascertainment of exposure, and sufficient follow-up duration. Robinson et al. [17] scored 8, with slightly lower points for the comparability domain. In contrast, studies by Markman et al. [14] and Wight et al. [16] scored 7 due to limitations in follow-up duration and cohort adequacy, which may impact the reliability of long-term outcome assessments. Overall, the majority of the studies demonstrated methodological rigor, enhancing confidence in their findings (Table 2).

**Table 2:** Quality assessment of cohort studies by the Newcastle-Ottawa Scale.

Study	Representativeness of the Exposed Cohort (1)	Selection of the Non-Exposed Cohort (1)	Ascertainment of Exposure (1)	Demonstration that Outcome of Interest was Not Present at Start of Study (1)	Compare Ability of Cohorts on the Basis of the Design or Analysis (2)	Assessment of Outcome (1)	Was Follow-Up Long Enough for Outcomes to Occur (1)	Adequacy of Follow-Up of Cohorts (1)	Total Quality Score (Out of 9)
Toniolo et al. [13]	1	1	1	1	2	1	1	1	9
Markman et al. [14]	1	1	1	1	2	1	0	0	7
Ho et al. [19]	1	1	1	1	2	1	1	1	9
Van et al. [15]	1	1	1	1	2	1	1	1	9
Wight et al. [16]	1	1	1	1	2	1	0	0	7
Robinson et al. [17]	1	1	1	1	1	1	1	1	8
Conte et al. [22]	1	1	1	1	2	1	1	1	9
Fudim et al. [18]	1	1	1	1	2	1	1	1	9

**Figure 2:**
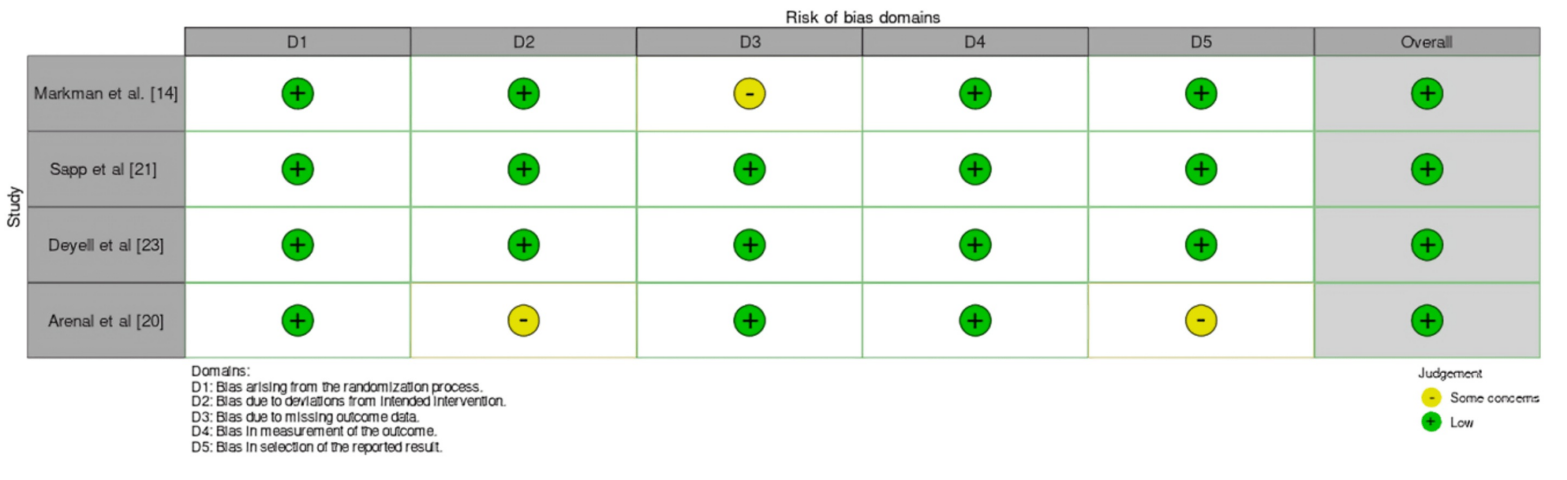
Quality assessment of randomized controlled trials by RoB 2.0.

Studies were assessed using the risk of bias tool, RoB 2.0 (The Cochrane Collaboration, London, United Kingdom), showing a low risk of bias across all domains: randomization process (D1), deviations from intended intervention (D2), missing outcome data (D3), outcome measurement (D4), and selection of reported results (D5). In the study by Markman et al., minor concerns were noted regarding missing outcome data, the study maintains a low risk of bias, ensuring reliable and trustworthy results [14]. Similarly, Sapp et al. [21] and Deyell et al. [23] demonstrated a low risk of bias in every domain, including proper randomization, intervention implementation, complete outcome data, accurate measurement, and unbiased reporting. These studies reflect strong methodological quality and reliable findings. In contrast, Arenal et al. [20] showed some concerns regarding deviations from the intended intervention (D2) and the selection of reported results (D5). While other domains were assessed as low risk, these concerns highlight the need to interpret the study’s findings carefully. Overall, the studies maintain solid methodological standards (Figure 2).

Discussion

With their distinct mechanistic benefits, the evaluated imaging-guided treatments showed significant effectiveness in lowering VT episodes and associated interventions. Computational ECG mapping and STAR are two examples of developments in accurate arrhythmogenic substrate localization. STAR treatment, which combines precise targeting of VT circuits with noninvasive irradiation, had a median VT episode reduction of 87%. Similarly, by improving radiation administration accuracy by colocalization with previous invasive mapping, computational ECG mapping considerably decreased defibrillator shocks (from 23 ± 12 to 0.67 ± 1.0; P < 0.001) [19].

The value of advanced imaging is further highlighted by emerging technologies like noninvasive electrophysiology-guided cardiac radioablation, which demonstrated improvements in five out of nine quality-of-life domains and reduced VT episodes from a median of 119 to 3 (P < 0.001) [17]. TcMS and CCT integrated with EAM offered novel approaches for the identification and management of arrhythmogenic substrates, with TcMS significantly reducing VT episodes when compared to sham treatments (4.5 vs. 10.7; P < 0.001) [14]. A diagnostic accuracy of 94.1% for the diagnosis of myocardial fibrosis was achieved by combining cardiac CT with EAM [22]. This feature is especially helpful for patients for whom MRI is not appropriate since it provides a good substitute for detecting arrhythmogenic substrates and directing ablation techniques.

In terms of safety and effectiveness, pharmacological methods differed greatly. When compared to traditional antiarrhythmic medications, procainamide demonstrated superior efficacy in reducing VT episodes (median reduction from 19 to 5.5; P < 0.05). However, it also had a significant incidence of side effects (8.8% experiencing hypotension or dyspnea), which in some cases required stopping therapy [13]. Sotalol and cibenzoline, on the other hand, have superior safety profiles; nevertheless, this evaluation did not include direct comparable data. According to Fudim et al., noninvasive treatments such as ultrasound-guided bilateral SGB have shown exceptional safety and effectiveness, with 45% of patients experiencing full VT suppression for 48 hours with few side effects [18]. These results demonstrate the possibility of including SGB in acute VT treatment regimens.

Escalated AAD therapy in patients with ischemic cardiomyopathy and VT resulted in worse outcomes compared to catheter ablation, with higher rates of severe complications and composite adverse outcomes [20,21]. Ablation demonstrated a 59.1% incidence of unfavorable outcomes compared to 68.5% with AAD escalation (HR 0.72, P = 0.04), particularly benefiting patients with slow VT [23]. These findings emphasize the limitations of AADs in managing recurrent VT, particularly in high-risk populations.

This systematic review's limitations include the heterogeneity of the included studies, which differed in sample size, intervention modalities, and design (RCTs, cohort studies, and clinical trials), making meta-analysis impossible and direct comparisons challenging. Potential selection bias may have been introduced by the exclusion of pertinent research due to the language limitation to English publications. The review focused on studies from the last 10 years, which may have overlooked seminal works predating this period, limiting historical context. Furthermore, the scope of findings in the context of changing therapeutic breakthroughs may be limited if research considering non-traditional medicines like gene therapy or sophisticated cardiac devices is excluded. The majority of the included research, especially the cohort studies, had small sample sizes, which limited how broadly the findings could be applied. The exclusion of case reports also limits the research evidence. Furthermore, the variability in reporting outcomes such as VT burden, ICD interventions, and quality-of-life measures across studies hindered comprehensive synthesis. Lastly, the lack of long-term follow-up data in several studies raises concerns about the durability and safety of certain interventions over time.

Large-scale, multicenter trials with standardized protocols should be conducted in the future to validate the long-term efficacy and safety of imaging-guided therapies like STAR, computational ECG mapping, and noninvasive radioablation, investigating the integration of advanced technologies such as artificial intelligence for precise detection of arrhythmogenic substrates and optimization of therapy, conducting comparative studies evaluating the cost-effectiveness and optimal combinations of imaging modalities with pharmacological or catheter-based interventions, and looking into patient-specific factors such as comorbidities and arrhythmia characteristics as these could lead to customized treatment plans, ultimately improving clinical outcomes and quality of life for patients with VT.

## Conclusions

This systematic review highlights the promising impact of advanced imaging and pharmacological therapies on key clinical outcomes in the management of critically ill patients with refractory VT. There were notable decreases in VT events, with some groups experiencing reductions of over 75% thanks to transcutaneous magnetic stimulation and STAR. Computational ECG mapping and noninvasive cardiac radioablation have shown significant decreases in ICD shocks, enhancing patient safety and quality of life. Furthermore, antiarrhythmic medication uses and hospital readmissions were considerably reduced by STAR treatment and ultrasound-guided SGB. The results are constrained by varied demographics, research heterogeneity, and small sample numbers. While a paucity of long-term evidence restricts judgments on efficacy and cost-effectiveness, adverse events such as hypotension (procainamide) and pneumonitis (STAR) draw attention to safety issues. In order to improve methods, evaluate safety, and investigate cost-effectiveness, larger, carefully planned studies with longer follow-ups are required. Critically sick VT patients may benefit from individualized therapy when imaging and pharmaceutical approaches are combined.
